# Dicyanopyrazine: 10th Anniversary in Photoredox Catalysis

**DOI:** 10.1002/tcr.202500134

**Published:** 2025-09-04

**Authors:** Zuzana Burešová, Filip Bureš

**Affiliations:** ^1^ Institute of Organic Chemistry and Technology Faculty of Chemical Technology, University of Pardubice Studentská 573 Pardubice 532 10 Czechia

**Keywords:** dicyanopyrazine, dithienoquinoxaline, photocatalysts, photoredox catalysis, structure‐property relationships

## Abstract

The last decade unveiled dicyanopyrazine as a purely organic photocatalyst capable of initiating a variety of unprecedented photoredox transformations. The latest discoveries also pointed to a facile Mallory‐type photocyclization of the catalyst to quinoxaline‐2,3‐dicarbonitrile derivative, which proved to be the active catalytic species. Its principal photochemical properties involve the absorption band covering the blue spectral region, a sufficiently long‐lived triplet, and the reversible first reduction accompanied by the formation of the corresponding radical anion. Hence, two‐photon photoredox catalysis via (consecutive) photoinduced electron transfer can be conveniently accomplished to either oxidize or reduce various substrates. This review summarizes the first synthetic attempts toward dicyanopyrazine catalyst, its further improvements, structural modifications, photochemical properties, and also covers the application of pyrazine‐2, 3‐dicarbonitirle and quinoxaline‐2,3‐dicarbonitrile‐based photocatalysts across the photoredox catalysis.

## Graphical abstract

Ten years of dicyanopyrazine photocatalysts in photoredox catalysis have been summarized. The historical background, syntheses, structural modifications, mechanism of action, and application across modern photochemical transformations are covered.

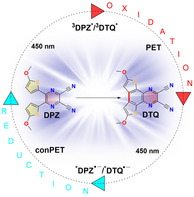

## 1 Introduction

Light gives life. Plants and flowers bend toward the sun to increase incident photon flux an essential prerequisite of efficient photosynthesis. Thanks to chlorophyll, plants can convert the energy of a photon into chemical energy and, subsequently, release the stored energy in the form of sugar to support the organism´s growth. Although O_2_ and CO_2_ are not the common accompanying products/reactants, photoredox catalysis utilizes the same principle by converting the energy of the visible light into the energy of a chemical bond. Since the pioneering experiments carried out by G. Ciamician in 1912,^[^ ^1^ ^]^ nowadays, photochemically generated radicals are being advantageously utilized in various chemical reactions. Such facile formation of organic radicals has truly revolutionized organic synthesis, as documented by the immense number of publications on photoredox catalysis published over the last 10 years. In general, a photocatalyst (PC) used in photoredox catalysis has the same function as chlorophyll in photosynthesis both assure absorption of the light and further utilization of the acquired energy. Historically, transition metal‐based PCs,^[^ ^2^ ^]^ polypyridyl complexes of ruthenium and iridium in particular, belong to the most explored and prominent group of PCs with superior properties and proven wide applications across organic synthesis.^[^ ^3^ ^]^ A combination of PCs with well‐known (transition) metal catalysts and enzymes in dual catalysis^[^ ^4^ ^]^ opens the possible applications of photoredox catalysis even further. Along with the coordination complexes with photoredox activity, pure organic PCs were also developed and used in photoredox reactions.^[^ ^5^, ^6^, ^7^ ^]^ In general, these represent less toxic and inexpensive alternatives to metal‐based PCs, with a comparable redox power. Xanthene and acridinium dyes, flavins, perylenediimides, and cyanoarenes are typical representatives. In principle, a substance utilizable as a PC must undergo either photoinduced electron transfer (PET) or energy transfer (EnT) to/from the substrate, reagent, or cocatalyst. The PC should further meet some essential requirements^[^ ^8^, ^9^ ^]^ such as: 1) absorption in the visible region of the spectra, 2) (maximal) overlap of its absorption band with the emission band of the used light source, 3) reversible redox properties enabling PET or EnT and a closed catalytic cycle, 4) high (photo)chemical stability and 5) low catalytic loading. Likewise, the standard catalysis, homo and heterogenous PCs^[^ ^10^, ^11^ ^]^ can be applied in the current photoredox catalysis, which significantly facilitates the recyclability of the given PC. In 2014,^[^ ^12^ ^]^ dicyanopyrazine (**DPZ**) was introduced as a novel PC, and since that, significant progress in its preparation, structural variations, mechanisms of action, and application across organic synthesis has been achieved. Due to the 10th anniversary of **DPZ**, we review its fundamental aspects herein.

## 2 Dicyanopyrazine (DPZ)

**Scheme 1:**
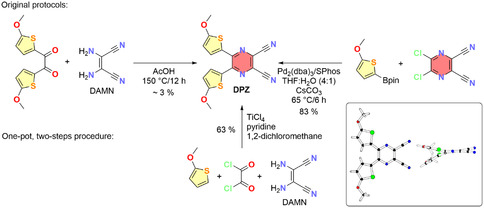
Synthetic pathways toward **DPZ** and its X‐ray molecular representation as an inset (CCDC 1 553 203).

The scientific interest in push–pull chromophores based on pyrazine‐2,3‐dicarbonitrile and isolobal benzene‐1,2‐dicarbonitrile can be dated back to 2012, when these scaffolds were utilized as powerful (heterocyclic) electron‐acceptor moieties to construct X‐shaped push–pull molecules.^[^ ^13^, ^14^ ^]^ Further structural variation of the *π*‐system and the appended peripheral electron‐donors resulted in 5,6‐bis(thiophen‐2‐yl)‐ and 5,6‐bis(5‐methoxythiophen‐2‐yl)‐substituted pyrazine‐2,3‐dicarbonitriles.^[^ ^12^ ^]^ The latter, denoted as **DPZ** (**Scheme** **1**), proved a very efficient PC in various benchmark photoredox transformations, but its preparation was tricky. Whereas the original acid‐catalyzed condensation of the 5,5′‐dimethoxy‐2,2′‐thenil with DAMN was very low‐yielding, the cross‐coupling was too demanding due to the necessary preparation of both starting components and the expensive catalytic system. Hence, a one‐pot two‐step procedure starting from commercially available 2‐methoxythiophene, oxalyldichloride, and DAMN, allowing a multigram preparation of **DPZ** in 63% yield within 2–3 h, has been developed.^[^ ^15^ ^]^ The X‐ray analysis of **DPZ** reveals a planar dicyanopyrazine fragment, outer orientation of both sulphur atoms of the methoxythiophene donors, and their out‐of‐plane arrangement with the dihedral angle of 8/24°. The side view in Scheme 1 implies that the overall nonplanar arrangement is forced by a repulsion of the inward‐oriented thiophene hydrogen atoms at the C3 positions.

## 3 DPZ's (Photo)Chemical Properties

**Figure 1:**
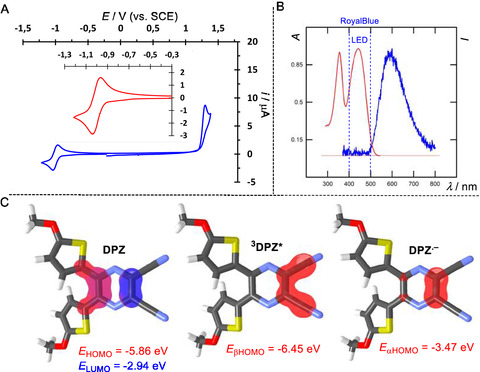
**DPZ**: A) Voltammogram in DMF at a scan rate of 100 mV s^−1^, the reversible reduction is shown as an inset, B) normalized UV–visible absorption (red) and fluorescence emission spectra (blue) along with a schematic emission band of the RoyalBlue LED, C) the density functional theory (DFT)‐calculated HOMO (red) and LUMO (blue) localizations in **DPZ**, the βHOMO spinorbital localization in ^**3**^ DPZ* and the αHOMO spinorbital localization in **DPZ** ^**•−**^.

**Figure 2:**
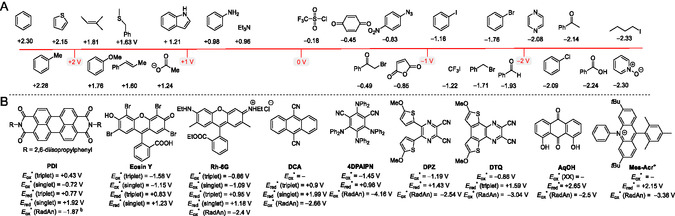
A) The reduction and oxidation potentials of selected organic substrates vs. SCE.^[^ ^73^ ^]^ B) The oxidation and reduction power of selected organic PCs.^[^ ^5^, ^74^, ^75^ ^–^ ^76^ ^]^

The **DPZ**'s cyclic voltammogram (**Figure** **1**A) points to a reversible reduction with the first reduction potential *E*_1/2(red)_ = –1.00 V, accompanied by an exchange of one electron, while the oxidation is an irreversible and multiple‐electron process (*E*_p(ox)_ = +1.30 V; **Table** **1**). This behavior indicates that **DPZ** forms stable radical anion and unstable radical cation, and as such, it may generally behave as a one‐electron oxidant suited for a reductive quenching cycle. Its UV–visible absorption spectrum (Figure 1B) contains two bands appearing at 442 and 355 nm with the low‐energy absorption feature arising from an intramolecular charge‐transfer transition as assumed from its D–π–A arrangement and the donor‐centered highest occupied molecular orbital (HOMO) and acceptor‐centered lowest unoccupied molecular orbital (LUMO) (Figure 1C). The CT‐band almost perfectly overlaps with the emission spectra of RoyalBlue light‐emitting diode (LED), a common light source used in the photoredox catalysis. **DPZ** is very weakly fluorescent with the emission maximum appearing at 590 nm and the quantum yield below 5% indicating the fluorescence as a minor **DPZ**'s relaxation pathway. The Stokes shift is moderate (5.675 cm^−1^) with the midpoint, denoted as the excited state energy (*E*_0,0_), equal to 2.46 eV. This imparts **DPZ** the excited state reduction potential *E*_red_* = +1.46 V. Hence, the oxidation power of excited **DPZ** is analogous to hydrogen peroxide or permanganate and comparable to organic PCs such as eosin Y, PDI, DCA, or 4DPAIPN, and **DPZ** can directly oxidize, for instance, amines, carboxylates, and selected aromatic *N*‐heterocycles (**Figure** **2**). Transient absorption spectroscopy suggests that **DPZ** forms a long‐lived triplet excited state (*τ* ≈ 15 μs) upon intersystem crossing (ISC) from the short‐lived singlet excited state; therefore, ^**3**^ DPZ* is considered the active species participating in PET. This is further supported by its low‐lying βHOMO spin orbital (–6.45 eV) spread over the cyano groups (Figure 1C), both are chemically susceptible to one‐electron reduction, accompanied by the formation of the corresponding radical anion **DPZ** ^**•−**^. Hence, ^**3**^ DPZ* is the catalytic species prone to accept one electron, which principally determines its one‐electron oxidation potential toward a substrate.

**Table 1:** Fundamental redox and optical properties of the discussed PCs. All data measured in DMF.^[^ ^30^ ^]^

Cat.	*E*_1/2(red)_ [V]a)	*E*_p(ox)_ [V]a)	*λ*_max_^A^ [nm eV^−1^]b)	*λ*_max_^F^ [nm eV^−1^]b)	*λ* ^F^ [%]b)	Stokes shift [cm^−1^ eV^−1^]b)	*τ* ^T^ [μs]c)	Triplet		Radical Anion
								*E*_0,0_ [eV]d)	*E*_red_* [V]e)	*E*_ox(RadAn)_* [V]f)
**DPZ**	–1.00	+1.30	442/2.81	590/2.10	<5	5675/0.71	14.6	2.46	1.46	–2.63
**DTQ**	–0.78	+1.63	467/2.66	571/2.17	<5	3900/0.49	29.6	2.42	1.64	–2.99

**Figure 3:**
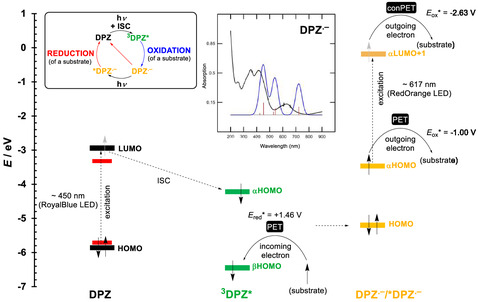
Energy level diagram showing the frontier and spin orbitals of **DPZ**, ^**3**^ **DPZ** ^*^, and **DPZ** ^**•−**^/*DPZ ^**•−**^ that are involved in the reductive quenching cycle and may accomplish oxidation/reduction of a substrate via PET and conPET. The experimental (black) and DFT‐calculated (red) absorption spectrum of **DPZ** ^**•−**^ is shown as the inset.

a) Measured by cyclic voltammetry (*v* = 100 mV s^−1^) vs. saturated calomel electrode (SCE).

b) Measured by the absorption and emission spectroscopy.

c) Lifetime of the triplet excited state measured by the transient absorption spectroscopy.

d) The excited state energy calculated as *E*_0,0_ = (*λ*_max_^A^ – *λ*_max_^F^)/2 + *λ*_max_^F^ (eV).

e) The excited state reduction potential calculated as *E*_red_* = *E*_1/2(red)_ + *E*_0,0_.

f) The excited state oxidation potential of the corresponding radical anion (RadAn) is estimated as *E*_ox(RadAn)_* = *E*_1/2(red)_ – *E*_0,0_(RadAn/RadAn*). The excited state energy *E*_0,0_(RadAn/RadAn*) was estimated as an onset of the absorption spectra of the corresponding radical anion (see Figure 3).

**Figure** **3** shows an energy level diagram summarizing the principal electron transfers within **DPZ**. The initial excitation of an electron from the HOMO to the LUMO can be easily accomplished by the blue light, affording the singlet excited state that quickly transforms into the triplet excited state via the ISC. The long‐lived triplet ^**3**^ DPZ* easily accepts an electron into the βHOMO, and the PET results in the oxidation of a substrate. The formed electron‐rich radical anion **DPZ** ^**•−**^ can transfer the lone electron from the αHOMO directly to a substrate in terms of its reduction, but the reduction power is limited to –1.00 V (as electrochemically determined *E*_1/2(red)_) and is often thermodynamically unfeasibility. However, dark‐colored **DPZ** ^**•−**^ (see the inset of Figure 3 for its experimental and calculated absorption spectra) can be excited to *DPZ ^**•−**^ and undergo consecutive PET (conPET, Figure 3). The reduction power of *DPZ ^**•−**^ is significantly enhanced (–2.63 V) and approaches the power of highly reducing alkaline metals. Moreover, it exceeds the power of known PCs such as PDI or Rh‐6 G (Figure 2). Utilization of *DPZ ^**•−**^ is partially hampered by its short lifetime (*τ* ≈ 1 ps), which rules out a bimolecular PET under diffusion control. The conPET from *DPZ ^**•−**^ is assumed to occur only with a preassociated substrate.^[^ ^16^ ^]^ In summary, despite **DPZ** obeying only the reductive quench cycle, it can be utilized either as an oxidant or a reductant. The latter is very often accomplished by pre‐oxidation of a sacrificial reagent such as *N*,*N*‐diisopropylethylamine (DIPEA), triethanolamine (TEOA), Hantzsch ester (HE), etc.

## 4 Structural Elaboration with DPZ

**Figure 4:**
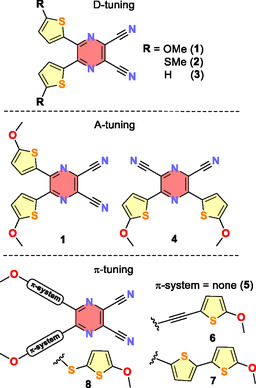
The structural modification of the original **DPZ** catalyst via D–, A–, and π–tuning.

**DPZ** possesses an X‐shaped push–pull arrangement comprising the dicyanopyrazine acceptor and methoxythiophene donors, and such D–π–A structure principally allows D–, A–, and π–tuning.^[^ ^17^ ^]^ Variation of the donor groups has been addressed by preparing a series of analogous MeO‐, MeS‐, and H‐substituted catalysts **1**–**3** (**Figure** **4**, **Table** **2**). Whereas replacement of MeO in **1** by MeS (**2**) did not affect the absorption maxima, the unsubstituted derivative **3** possesses the longest‐wavelength absorption maxima, shifted to 379 nm, out of the emission window of blue LED. Hence, despite the highest excited state reduction potential (–1.79 V), the catalytic activity of **3** is lower than that of **1** and **2**. Both these catalysts showed similar catalytic activity in benchmark cross‐dehydrogenative coupling (CDC) and annulation reactions, but **1** is preferred due to its facile and straightforward synthesis.^[^ ^18^, ^19^ ^]^ The principal variation of the acceptor moiety may involve pyrazine positional isomers, namely pyrazine‐2,3‐dicarbonitrile (**1**), pyrazine‐2,6‐dicarbonitrile (**4**), and pyrazine‐2,5‐dicarbonitrile, the latter proved synthetically unfeasible. When comparing **1** and **4**, both are reversibly reduced at around –1.15 V, but the absorption maxima of **4** are blue shifted to 389 nm, which is prohibitive to an efficient excitation with the blue LED. An eventual full replacement of the pyrazinedicarbonitrile acceptor moiety by imidazoledicarbonitrile has detrimental effects on the catalytic activity as well.^[^ ^19^ ^]^

**Table 2:** Fundamental redox and optical properties of the modified DPZ catalysts.

Cat.	*E*_1/2(red)_ [V]a)	*λ*_max_^A^ [nm eV^−1^]b)	Excited state	
			*E*_0,0_ [eV]c)	*E*_red_* [V]d)
**1, DPZ**	–1.14	440/2.82	2.50	+1.36
**2**	–1.01	443/2.79	2.22	+1.21
**3**	–1.12	379/3.27	2.91	+1.79
**4**	–1.15	389/3.19	2.70	+1.55
**5**	–1.53	278/4.46	4.00	+2.47
**6**	–0.80	459/2.70	2.59	+1.82
**7**	–0.95	511/2.43	–	–
**8**	–	279/4.44	–	–

a) Measured by cyclic voltammetry in ACN (v = 100 mV s^−1^).

b) Measured in DCM.

c) Excited state energy calculated as *E*_0,0_ = *λ*_max_^A^ – *λ*_max_^F^.

d) Excited state reduction potential calculated as *E*_red_* = *E*_1/2(red)_ + *E*_0,0_.

The tuning through the *π*‐system can be demonstrated with PCs **5**–**8** (Figure 4). As can be seen, none (**5**) or a nonconjugated sulfidic *π*‐linker (**8**) shifted the absorption maxima deeply in the UV area (278 and 279 nm), which resulted in no catalytic activity. The twisted structure of **DPZ** has been planarized by introducing additional acetylenic spacers as in **6**, which shifted the ground‐state first reduction potential to positive values and increased the excited state reduction potential to +1.82 V. An insertion of thiophen‐2,5‐diyl moieties (**7**) shifted the absorption maxima bathochromically to 511 nm and fully suppressed the emissive properties. Anyway, the catalytic activity of **6** and **7** in benchmark reactions was below the activity of **DPZ**. Starting from available 5,6‐dichloropyrazine‐2,3‐dicarbonitrile or 3,5‐dichloropyrazine‐2,6‐dicarbonitrile, the aforementioned derivatives are accessible either via Suzuki–Miyaura and Sonogashira cross‐coupling reactions or nucleophilic substitution. Till today, the photoredox catalytic activity of **DPZ** is superior, and only methylsulfanyl derivative **2** can be considered close to efficient.

## 5 Photoredox Transformations Mediated by DPZ

### 5.1 Initial Reactions

**Scheme 2:**
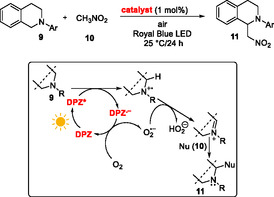
The first aza‐Henry reaction catalyzed by **DPZ**, including the plausible catalytic cycle.

In 2014, **DPZ** was first examined in the CDC (aza‐Henry) reaction^[^ ^20^ ^]^ between *N*–phenyltetrahydroisoquinoline (THIQ) and nitromethane, affording the desired product smoothly and quantitatively (**Scheme** **2**).^[^ ^12^ ^]^ This α‐functionalization has been successfully applied to (a)cyclic amines and a variety of C–nucleophiles such as nitroalkanes, acetone, 5‐methyl‐2‐fenyloxazole‐4(5* H*)‐on, diethyl phosphite, and trimethylsilylcyanide, affording target compounds **11** in high yields (76%–95%). A general **DPZ** loading was 0.1 mol%, but for some transformations, 0.01 mol% of **DPZ** proved sufficient. The plausible reaction mechanism obeys the reductive quench cycle, including PET from the starting amine **9** to the excited DPZ*, and the formed **DPZ** ^**•−**^ is subsequently regenerated by a reaction with nitromethane or oxygen. The formed superoxide O_2_^•‐^ further deprotonates the ammonium radical cation to the corresponding iminium salt, which undergoes a nucleophilic attack to **11**. In a similar way, **DPZ** can also be employed in an aerobic dehydrogenation of cyclic aminoketones yielding 2,3‐dihydro‐4‐pyridones and 4‐quinolones.^[^ ^21^ ^]^

**Scheme 3:**
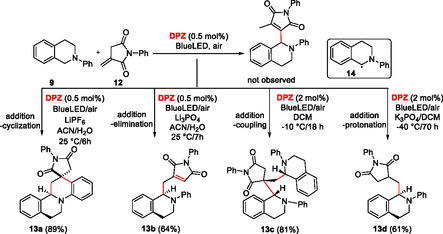
The chemodivergent cascade reaction of THIQ and itaconimides mediated by **DPZ**.

**Scheme 4:**
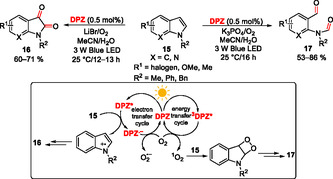
**DPZ**‐mediated aerobic oxygenation of indoles.

Surprisingly, *N*‐substituted itaconimides **12** reacted with THIQs **9** differently in a **DPZ**‐initiated chemodivergent cascade (**Scheme** **3**).^[^ ^22^ ^]^ The products **13a**–**d** were formed via a sequence involving addition‐cyclization, addition‐elimination, addition‐coupling, and addition‐deprotonation. Itaconimides possess an activated exocyclic double bond and an enolizable amide function, imparting both electrophilic and nucleophilic character of **12**. Hence, the radical **14** formed in the reductive quench cycle from **9** may undergo the aforementioned transformations resulting in **13a**–**d** based on the applied reaction condition (catalyst loading, solvent, additive, and temperature). *N*‐Pendants and substitution of both starting THIQ (**9**) and itaconimide (**12**) were screened. Chemodivergence using **DPZ** photocatalysis has been further demonstrated in an aerobic oxygenation of 2‐substituted indoles **15**, affording either isatines **16** or formanilides **17** (**Scheme** **4**).^[^ ^23^ ^]^ pH proved to be a chemoselective switch distinguishing both reaction pathways. Whereas isatines **16** originate from the **DPZ**‐initiated reductive quench cycle affording indole radical cation, formanilides **17** are formed upon energy transfer from ^**3**^ **DPZ** * (*E*_T_ = 46.6 kcal mol^−1^) to ^3^O_2_ → ^1^O_2_ (*E*(1Δ–3Σ) = 22.5 kcal mol^−1^), yielding a dioxetane derivative, which further undergoes hydrolysis to **17**. A similar visible light‐driven aerobic oxidation of benzylic sp^3^ C—H bonds utilizing a cooperative catalysis between **DPZ** and *N*‐hydroxyphthalimide (NHPI) has been developed by Jiang et al. in 2016.^[^ ^24^ ^]^ A variety of carbonyls can be efficiently prepared from benzylic methylenes and benzylic alcohols in this way.

### 5.2 α‐Functionalization

**Scheme 5:**
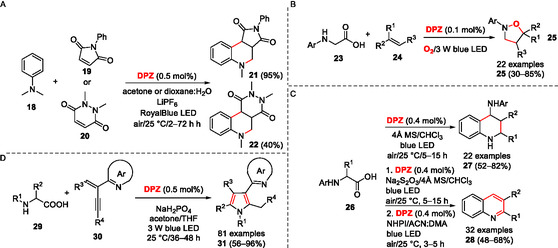
α‐Functionalization of amines and α‐amino acids under **DPZ** photocatalysis and utilization of the resulting radical species in valuable organic transformations (MS denotes molecular sieves).

**Scheme 6:**
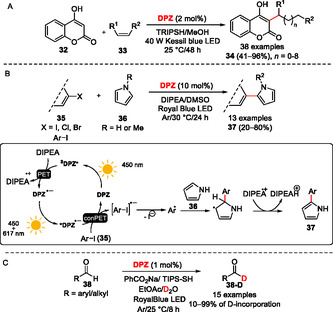
α‐Functionalization of hydroxycoumarine, photochemical cross‐coupling, and deuteration promoted by **DPZ**.

α‐Functionalization of amines and related substrates (Figure 2) can be generally easily achieved via photoredox catalysis, and the initial reactions (Scheme 2, 3) showed that **DPZ** can initiate these transformations efficiently. In addition, **DPZ** mediates also formation of a radical from *N*,*N*‐dimethylaniline (**18**), which adds to an electron‐poor double bond of *N*–phenylmaleimide **19** or 1,2‐dimethyl‐1,2‐dihydropyrazine‐3,6‐dione **20**. Upon subsequent cyclization facilitated by LiPF_6_, biologically relevant pyrrolo/pyridazinoquinolinediones **21**/**22** can be synthesized (**Scheme** **5**A).^[^ ^18^ ^]^ A similar strategy can also be applied to readily available α‐amino acids. For instance, ^**3**^ **DPZ** * may oxidize *N*‐arylglycine **23** with a concomitant decarboxylation. The formed α‐aminoalkyl radical adds to alkenes **24**, and the products are subsequently trapped by superoxide under formation of isoxazolidines **25** (Scheme 5B).^[^ ^25^ ^]^ Starting from *N*‐arylamino acids **26**, a cascade aerobic decarboxylative Povarov and oxidative dehydrogenation reaction under **DPZ** photocatalysis afforded 4‐aminotetrahydroquinolines **27**, which may be further oxidized to 2,3‐disubstituted quinolines **28** with the aid of **DPZ**/NHPI (Scheme 5C).^[^ ^26^ ^]^ The reaction sequence involves again **DPZ**‐mediated oxidation of **26** to the radical cation, decarboxylation, dehydrogenation affording imine/enamine, their cyclization to **27**, and eventual oxidation with **DPZ**/NHPI to **28**. *N*‐Aryl α‐amino acids **29** and 2‐substituted 1,3‐enynes **30** were utilized as starting compounds for the construction of azaarene‐substituted highly functionalized pyrroles **31** (Scheme 5D).^[^ ^27^ ^]^ The reaction is initiated by oxidation/decarboxylation of the α‐amino acid **29** by ^**3**^ **DPZ** *, and it continues by an addition of the formed α‐aminoradical to 1,3‐enyne **30** and oxidation of the resulting intermediate to **31**. The reaction showed a broad substrate scope and the yields up to 96%. **DPZ** also proved efficient in promoting α‐functionalization of ketones as demonstrated on hydroxycoumarine **32** and olefins **33** (**Scheme** **6**A).^[^ ^28^ ^]^ Surprisingly, the reaction mechanism proposes **DPZ**'s oxidative quench cycle and formation of **DPZ** ^**•+**^, combined with 2,4,6‐triisopropylbenzenethiol hydrogen atom donor and methanol as a solvent, significantly facilitates and makes the oxidation of **32** thermodynamically feasible. The hydroxy group of **32** proved to be essential for the reaction affording **34** with complex R^1^ and R^2^ pendants.

### 5.3 Radical Cross‐Coupling

As demonstrated above (Figure 3), **DPZ** may principally act as a one‐electron oxidant (^**3**^ **DPZ** *) or reductant (**DPZ** ^**•−**^). The latter has been utilized in the conPET process of a photochemical cross‐coupling between various halogen (hetero)aromatics **35** and pyrrole **36** (Scheme 6B).^[^ ^29^ ^]^ Involving DIPEA as a sacrificial electron donor, this **DPZ**‐catalyzed reaction allows a facile construction of bi(hetero)aryls **37**, including biologically relevant atorvastatin or benzo[*c*][1,2,5]thiadiazole‐based semiconductors.

### 5.4 Deuteration

Isotopically‐labeled compounds, especially nonradioactive deuterium‐labeled substances, are extensively used across the pharmaceutical industry. In this respect, we have demonstrated a facile deuteration of various aldehydes **38** using **DPZ** photoredox catalyst (Scheme 6C).^[^ ^30^ ^]^ The reaction sequence is mediated by ^**3**^ **DPZ** *, which is capable of oxidizing benzoate and, jointly with TIPS‐SH, participates in the hydrogen atom transfer from the starting aldehyde **38**. The crucial aroyl radical R(O=)C· is deuterated using D_2_O and TIPS‐SH/D (deuterium transfer catalyst) to **38‐D**. Excellent D‐incorporation up to 99% can be achieved under **DPZ** catalysis.

### 5.5 Stereochemical Transformations

**Scheme 7:**
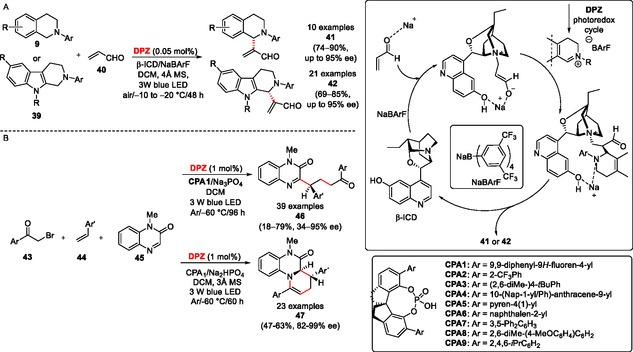
The dual photoredox and asymmetric catalysis involving **DPZ** and β‐isocupreidin or chiral phosphonic acid.

Very often, a stereogenic center(s) is(are) being formed during photoredox transformations, but a stereochemical control over the formation and further reaction of the formed radical (ions) is generally very challenging.^[^ ^31^, ^32^ ^]^ However, several elegant asymmetric strategies involving **DPZ** were recently demonstrated in the literature, mostly by the working group of Z. Jiang. A cooperative photoredox and asymmetric catalysis involving **DPZ** PC, β‐isocupreidin (β‐ICD) chiral Lewis base, and NaBArF as cocatalyst allows enantioselective aerobic olefination of THIQs **9** and tetrahydro‐β‐carbolines (THCs) **39** with acrolein **40** (**Scheme** **7**A).^[^ ^33^ ^]^

Analogously to the oxidation of THIQs shown in Scheme 2, the resulting iminium salt bearing bulky BArF^‐^ anion reacts with the β‐ICD‐preassociated **40** in terms of a nucleophilic attack, while β‐ICD principally discriminates the asymmetric induction and the attained optical purity of **41** and **42**. Besides β‐ICD, a combination of **DPZ** and a chiral phosphoric acid (CPA) proved to be another very useful strategy for a photocatalytic asymmetric synthesis. Jiang et al. have shown that linear or cyclic products **46** and **47** can be obtained via a radical cascade starting from 2‐bromo‐1‐arylethan‐1‐ones (**43**), styrenes (**44**), and quinoxalin‐2(1* H*)‐ones (**45**) (Scheme 7B).^[^ ^34^ ^]^ Whereas the formation of **46** or **47** can be directed via modulating pH of the environment (Na_3_PO_4_ vs. Na_2_HPO_4_), the asymmetric induction has been achieved with the aid of **CPA1** bonding **45** and thus discriminating the attack of the radical formed from **43** and **44** within the **DPZ**‐mediated photoredox cycle.

### 5.6 Asymmetric Transformations Utilizing α‐Amino Acids

**Scheme 8:**
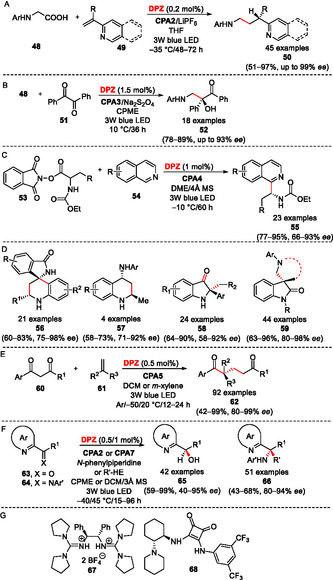
Representative applications of the **DPZ**‐mediated cooperative photoredox and asymmetric catalysis with optically active cocatalysts CPA, **67**, and **68**. See Scheme 7 for the structure of the particular CPA derivatives.

The aforementioned α‐functionalizations may also principally involve α‐amino acids, and Jiang et al. have demonstrated that a combination of **DPZ**/CPA‐mediated photoredox/asymmetric organocatalysis represents a useful strategy toward enantiomerically enriched products with a very broad applicability (**Scheme** **8**). For instance, *N*‐Aryl glycines **48** undergo a **DPZ**‐mediated decarboxylation affording *N*‐arylaminoalkyl radicals that enantioselectively add to 2‐vinylpyridines/2‐vinylquinolines **49** (azaarenes in general)^[^ ^35^ ^]^ coordinated by **CPA2**, providing an access to valuable chiral 3‐(2‐pyridine/quinoline)‐3‐substituted amines **50** (Scheme 8A).^[^ ^36^ ^]^ Similar asymmetric reactions have recently been reported for β‐ and *γ*‐substituted azaarenes structurally related to **49**,^[^ ^37^ ^]^ while photochemically generated radicals from (α, β‐unsaturated) ketones/imines and amides reacted with **49** in terms of an enantioselective cross‐coupling and asymmetric deoxygenative functionalization, respectively.^[^ ^38^, ^39^, ^40^ ^]^ **DPZ** proved to be the PC of choice, smoothly affording the desired radicals, either via oxidation of the amine or reduction of the ketone/amide. Glycine‐originated *N*‐arylaminoalkyl radicals also stereoselectively add to 1,2‐diketones **51** under **DPZ**/**CPA3** cocatalysis, affording enantiomerically enriched α‐hydroxyketones **52** (Scheme 8B).^[^ ^41^ ^]^ **DPZ**‐reductive quench cycle affords α‐aminoalkyl radicals also from α‐amino acid redox‐active esters **53** (RAEs) that undergo enantioselective Minisci‐type addition to isoquinolines **54** toward **55** in the presence of **CPA4** (Scheme 8C).^[^ ^42^ ^]^ α‐Aminoalkyl radicals generated via *DPZ‐mediated oxidation and decarboxylation of α‐amino acids were further utilized in enantioselective Povarov reaction, furnishing various valuable products such as isoindolin‐1‐ones **56** containing a 3,3‐spiro‐tetrahydroquinoline‐based stereocenter and 4‐amino‐2‐methyltetrahydroquinolines **57**, see Scheme 8D.^[^ ^26^, ^43^ ^]^ The asymmetric induction has been controlled using **CPA5** and **CPA6**, respectively. Under CPA/**DPZ** cocatalysis, *N*‐aryl amino acids can be further radically cross‐coupled with α‐bromoketones, affording enantioenriched β‐aminoketones.^[^ ^44^ ^]^ For further reading on the synthetic utilization of α‐aminoalkyl radical in visible light photoredox catalysis, the reader is referred to a review article by Nakajima et al.^[^ ^45^ ^]^ Structurally related and optically pure indolin‐3‐ones **58** and chiral 3‐aminomethylene‐3‐substituted oxindoles **59** are accessible via **DPZ**/**CPA5**‐mediated aerobic oxidation/semipinacol rearrangement and PET/radical coupling, respectively (Scheme 8D).^[^ ^46^, ^47^ ^]^

### 5.7 Reactions of (Di)Ketones and Azaarenes

The carbonyl compounds, as well as α‐substituted nitrogen‐containing heterocycles, represent popular scaffolds for the photochemical functionalization. For instance, Dexter energy transfer from the excited **DPZ** to chiral pyrenyl‐incorporated co‐sensitizer **CPA5** has been demonstrated as a dual catalytic system with enhanced redox properties capable of initiating asymmetric formal deMayo reaction on **60** (Scheme 8E).^[^ ^48^ ^]^ Such a catalysis platform enables otherwise thermodynamically unfeasible transformation of 1,3‐diketones **60** and variously 1,1‐disubstituted olefins **61** into valuable 1,5‐diketones **62** bearing a stereogenic center. Besides the stereoselective addition to 1,2‐diketones (Scheme 8B), **DPZ** may also participate in an enantioselective reduction of azaarene‐based ketones/imines **63**/**64**, provided a sacrificial amine (*N*–phenylpiperidine or R’‐HE) and **CPA2**/**CPA7** are present in the reaction mixture (Scheme 8 F).^[^ ^49^, ^50^ ^]^ Alcohols **65** and amines **66** with the optical purity up to 97% *ee* can be smoothly photochemically prepared this way. Surprisingly, a formation of the latter is rationalized via an oxidative quench cycle of **DPZ**. Prochiral azaarene ketones **63** and structurally related α‐chloroderivatives further undergo reduction/dehalogenation and subsequent asymmetric deuteration under CPA/**DPZ** cocatalysis, see also Scheme 6C for an analogy.^[^ ^51^ ^]^ In addition to the **DPZ**/CPA‐mediated photoreduction shown in Scheme 8F, 1,2‐diketones (e.g., benzil **51**, Scheme 8B) or related α‐keto ketimines can be stereoselectively reduced via enantioselective protonation to α‐hydroxy/amino ketones.^[^ ^52^ ^]^ The **DPZ**‐initiated reaction is stereochemically controlled with quinidinium salt **67** (Scheme 8G), but the concept of enantioselective protonation coupled with a radical cross‐coupling has recently also been extended to the **DPZ**/**CPA8** combination.^[^ ^53^ ^]^ An example of a cooperative photoredox/asymmetric catalysis involves the arylation of benzofuran‐2(3* H*)‐ones with substituted naphthols catalyzed by **DPZ** along with squaramide **68** (Scheme 8G).^[^ ^54^ ^]^ or a recent **DPZ**‐initiated three‐component reaction of cyanoazaarenes with 4‐substituted 1,4‐dihydropyridines and α‐boronate‐α‐substituted olefins.^[^ ^55^ ^]^

### 5.8 Photocyclizations

**Scheme 9:**
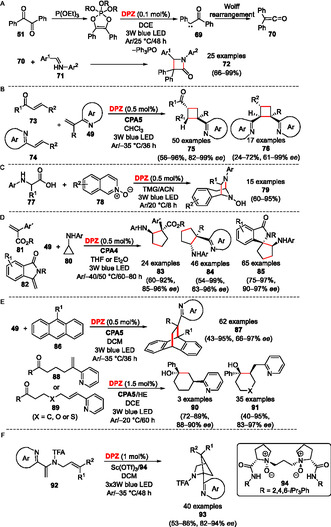
Photochemical cycloaddition reactions mediated by **DPZ**.

Photocycloaddition reactions represent one of the most explored and important photochemical processes utilized in organic synthesis.^[^ ^56^, ^57^ ^]^ In most cases, the cycloaddition can be accomplished as [2 + 2], [3 + 2], and [4 + 2] reactions, and **DPZ** proved to be a highly active PC allowing these transformations. For instance, in the presence of triethylphosphite, benzil **51** can be activated to 1,3,2‐dioxaphosphole, which undergoes **DPZ**‐mediated photoredox deoxygenation to carbene **69** and subsequent Wolff rearrangement to diphenylketene **70** (**Scheme** **9**A). The latter proved to be a sufficiently reactive intermediate for the Staudinger synthesis of β‐lactams **72**.^[^ ^58^ ^]^ Jiang has also demonstrated that under **DPZ**/**CPA5** cooperative catalysis, the cyclobutadiene ring in **75**/**76** can be enantioselectively constructed from enones/alkenylazaarenes **73**/**74** and vinylazaarenes **49** (Scheme 9B).^[^ ^59^ ^]^ A **DPZ**‐catalyzed [3 + 2] cycloaddition can be accomplished with *N*‐aryl α‐amino acids **77** and isoquinoline *N*‐oxides **78**, affording diazabicyclo[3.2.1]octanes **79** in chemical yields ranging from 60%–95% (Scheme 9C).^[^ ^60^ ^]^ Cyclopropylamines **80** were utilized as suitable 1,3‐synthons in asymmetric intermolecular [3 + 2] photocycloaddition with various olefins, e.g., 2‐aryl acrylates **81**, vinylazaarenes **49**, or 3‐methylene‐isoindolin‐1‐one **82** (Scheme 9D).^[^ ^61^, ^62^, ^63^ ^]^ The **DPZ**‐initiated reactions carried out in the presence of selected CPAs afforded valuable cyclopentylamines **83**–**85** with the yield and the optical purity up to 99% and 97% *ee*, respectively. Catalytic efficiency of **DPZ** in [4 + 2] cycloaddition can be demonstrated by enantioselective dearomative reaction of vinylazaarenes **49** with anthracenes **86** (Ref. [64]) or Beckwith–Enholm intramolecular cyclization of **88**/**89** toward **87** and **90**/**91** (Scheme 9E).^[^ ^65^ ^]^ Very recently, **DPZ**, along with the optically active *N*‐oxide **94** and Sc(OTf)_3_, was shown to initiate enantioselective intramolecular [2 + 2] photocycloaddition toward azabicyclo[2.1.1]hexanes **93** (Scheme 9F).^[^ ^66^ ^]^

### 5.9 Photochemical Deracemization and Protonation

**Scheme 10:**
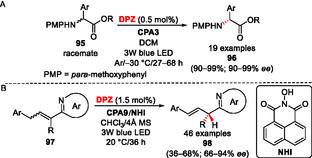
Deracemization and isomerization are achieved by a dual catalytic system, **DPZ**/CPA.

Converting a racemate into a single enantiomer, deracemization, belongs to fundamental catalytic tasks.^[^ ^67^ ^]^ Hence, a reagent‐free sequential photoredox and asymmetric catalysis with **DPZ** and **CPA3** was employed to deracemize α‐amino esters **95** (**Scheme** **10**A).^[^ ^68^ ^]^ The reaction mechanism involves *DPZ‐mediated oxidation of the starting amine to the corresponding radical cation (PET), deprotonation, subsequent single electron transfer from **DPZ** ^**•−**^, and the resulting C‐anion of **95** is finally enantioselectively protonated with **CPA3**. This methodology offers near quantitative yields and the enantiomeric purities ranging from 90–99%. A similar strategy has also been applied to α‐halogenketones that undergo a straightforward dehalogenation under **DPZ** catalysis and subsequent asymmetric protonation mediated by **68** and a cyclic secondary amine.^[^ ^69^ ^]^ Coupling the photoredox catalysis (**DPZ**), enantioselective protonation (**CPA9**), and hydrogen atom transfer with *N*‐hydroxyimide (**NHI**) allowed asymmetric isomerization of alkenylazaarenes **97** toward **98** (Scheme 10B).^[^ ^70^ ^]^

## 6 Photofading of DPZ, Dawn of DTQ

**Figure 5:**
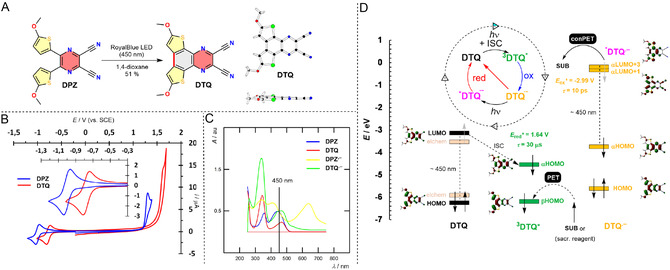
A) **DTQ**:The Mallory‐type photocyclization of **DPZ** to **DTQ** and its X‐ray structure, B) the voltammograms of both catalysts in DMF at a scan rate of 100 mV s^−1^, C) the absorption spectra of **DPZ**/**DTQ** and **DPZ** ^**•‐**^/**DTQ** ^**•‐**^ in DMF, D) the **DTQ**'s plausible mechanism of actions.

**Scheme 11:**
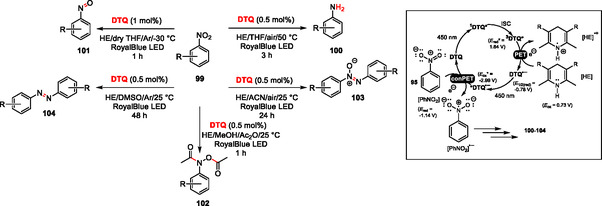
Chemodivergent photoreduction of nitroaromatics toward various reduction stages via modulation of the reduction power of *DTQ ^**•‐**^.

Our recent discovery pointed to a facile Mallory‐type photocyclization of **DPZ** upon irradiation with a blue light, affording 6,9‐dimethoxydithieno[2,3‐*f*:3',2'‐*h*]quinoxaline‐2,3‐dicarbonitrile (**DTQ**; **Figure** **5**A).^[^ ^71^ ^]^ In contrast to **DPZ** (Scheme 1), the **DTQ**'s fused *π*‐system is completely flat, which shifts its reduction to a more positive potential (*E*_1/2(red)_ = –0.78 V; Figure 5B), slightly red shifts the absorption maxima (*λ*_max_^A^ = 467 nm; Figure 5C), reduces the Stokes shift (structural reorganization upon excitation), makes its triplet excited state longer‐lived (*τ* ^T^ = 29.6 μs), and also more potent oxidant (*E*_red_* = +1.64 V; Table 1). Compared to **DPZ** and other state‐of‐the‐art organic PCs (Figure 2), **DTQ** withstands prolonged irradiation (>72 h) without significant photobleaching. On the contrary, the more positive first reduction potential also implies that **DTQ** ^**•−**^ is less powerful reductant compared to **DPZ** ^**•−**^, but its longest‐wavelength absorption maximum remained localized at around 450 and thus **DTQ** ^**•−**^ can be excited to a shortly‐lived double excited state *DTQ ^**•−**^ (*τ* ≈ 10 ps) with a single LED source. The blueshifted spectrum of **DTQ** ^**•−**^ (as compared to **DPZ** ^**•−**^) accounts for its extreme reduction power *E*_ox(RadAn)_* = –2.99 V, which is not far from that known for alkaline metals (e.g., –3.04 V for Li). Hence, **DTQ** may be principally employed as both a one‐electron oxidant/reductant but with significantly improved photoredox properties over **DPZ** (Figure 5D). As a matter of fact, the prompt formation of **DTQ** from **DPZ** under blue light irradiation in various solvents^[^ ^71^ ^]^ suggests that in situ formed **DTQ** is the likeliest active catalytic species in the aforementioned **DPZ**‐initiated reactions (Scheme 1, 2, 3, 4, 2, 3, 4, 5, 10). Its quick formation can also be monitored by thin layer chromatography or gas chromatography/mass spectrometry. This can be demonstrated by employing *DTQ ^**•−**^ in a chemodivergent photoreduction of nitroaromatics **99** to amino (**100**), nitroso (**101**), bis‐(*N*,*O*‐diacetyl)‐*N*‐arylhydroxylamine (**102**), azoxy (**103**), and azo (**104**) derivatives via conPET process (**Scheme** **11**). Whereas the loading of **DPZ** is limited to 4.5 mol%, **DTQ** can initiate the reaction even at 0.5 mol% loading and provides the desired product significantly faster. More importantly, the reduction power of *DTQ ^**•−**^ can be easily modulated by the used solvent, temperature, catalyst loading, and the reaction conditions to capture the particular reduction stage **100**–**104**.

**Scheme 12:**
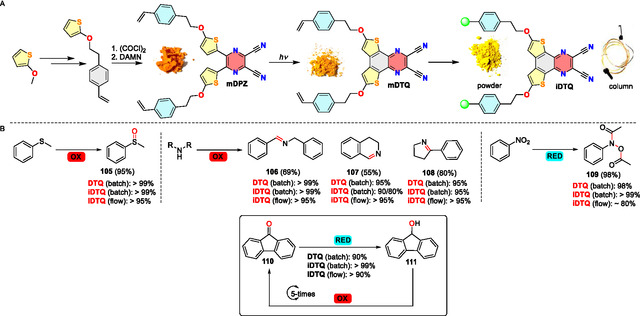
A) Synthetic pathway toward polymeric catalyst **iDTQ**, B) Homo/heterogeneous oxidation and reduction processes catalyzed by **DTQ** and **iDTQ** either in batch or under continuous‐flow conditions.

With an efficient **DTQ** catalyst in hand, we have undertaken further steps toward its immobilization via a straightforward copolymerization approach as outlined in **Scheme** **12**A.^[^ ^72^ ^]^ Starting from 2‐methoxythiophene, we have replaced the methoxy group by 2‐styrylethoxy pendant, employed the aforementioned one‐pot/two‐step procedure to **mDPZ**, which underwent Mallory photocyclization and subsequent copolymerization with styrene to **iDTQ**. This polymeric catalyst, prepared either as a powder or a flexible monolithic column, possesses essentially the same properties as homogenous **DTQ** and can be practically employed either in batch or continuous‐flow heterogeneous photoredox processes (Scheme 12B).

A comparison of **DTQ** and **iDTQ** in homo/heterogeneous oxidation of thioanisole to sulfoxide **105** and various amines to imines **106**–**108**, along with the reduction of nitrobenzene to hydroxylamine **109**, clearly indicates high efficiency of both catalysts. The versatility and robustness of **iDTQ** are further supported by a cyclic reduction ↔ oxidation process with 9* H–*fluoren‐9‐one (**110**) and 9* H*–fluoren‐9‐ol (**111**), which can be accomplished repeatedly with the recycled **iDTQ** without compromising its catalytic efficiency.

## 7 Summary and Outlook

Dicyanopyrazine was introduced in 2014 and has completed the decade as a powerful purely organic PC with the absorption band localized in the blue region of the spectra. Up to now, more than 20 dicyanopyrazine derivatives have been developed, but the original **DPZ** remains the most active PC. However, the recent finding disclosed a facile Mallory‐type photocyclization of **DPZ** to **DTQ**, which is the likeliest active catalytic species in **DPZ**/**DTQ**‐initiated photoredox transformations described in the literature. The reversible one‐electron reduction principally implies a reductive quenching cycle via the triplet excited state behaving as a powerful one‐electron oxidant. However, the formed **DPZ** ^**•−**^/**DTQ** ^**•−**^ radical anions can be utilized, upon double excitation, as one‐electron reductants. Hence, one/two‐photon photoredox catalysis can be accomplished either via PET or conPET processes. The portfolio of **DPZ**/**DTQ**‐initiated photoredox reactions is rich and includes the formation of C—C, C—N, C—P, and C—D bonds, various cascade reactions and cyclizations, while the recent reports also demonstrated that enantioselective transformations are feasible. **DPZ**/**DTQ** PCs were applied in more than 30 different photochemical transformations so far; their catalytic loading/reaction time can be reduced down to 0.01 mol%/20 min, and the attained yields and *ee*s are up to 99%. The homogeneous reactions are further complemented with polystyrene‐immobilized **iDTQ** catalyst, allowing the first heterogeneous photochemical transformations carried out either under batch or continuous‐flow conditions with the aid of a flexible polymeric column. This significantly enhances the recyclability and reuse of the catalyst. The developed photochemical protocols were demonstrated on industrially, pharmaceutically, or biologically relevant substrates, which further emphasizes their synthetic utility.

The recent reports indicate that the catalytic efficiency of **DTQ** significantly exceeds that of the original **DPZ**. Considering **DTQ**, respectively *DTQ ^**•−**^, as a very powerful reductant, we anticipate its further applications across various valuable reduction processes, which will complement the reported oxidations. A one‐pot combination of substrate oxidation (PET) and subsequent reduction (conPET) is another challenging task that deserves further exploration. **iDTQ** also opens a new venue toward heterogeneous photochemical processes, and especially heterogeneous asymmetric reactions would be very challenging.

In summary, the last decade with **DPZ** has been very fruitful, and we look forward to the upcoming decade with (**i**)**DTQ**.

## Conflict of Interest

The authors declare no conflict of interest.

## Author Contributions

**Zuzana Burešová**: writing—original draft, writing—review and editing. **Filip Bureš**: writing—review and editing.

## Data Availability

Data sharing is not applicable to this article as no new data were created or analyzed in this study.
